# Electrocardiographic criteria which have the best prognostic significance in hypertensive patients with echocardiographic hypertrophy of left ventricle: 15‐year prospective study

**DOI:** 10.1002/clc.23402

**Published:** 2020-06-03

**Authors:** Dragan B. Djordjevic, Ivan S. Tasic, Svetlana T. Kostic, Bojana N. Stamenkovic, Milan B. Lovic, Nikola D. Djordjevic, Goran P. Koracevic, Dragan B. Lovic

**Affiliations:** ^1^ University of Nis Medical Faculty Nis Serbia; ^2^ Institute Niska Banja Nis Serbia; ^3^ Clinical Center Nis Nis Serbia; ^4^ Singidunum University School of Medicine Clinic for Internal Diseases Intermedica Nis Serbia; ^5^ Veterans Affairs Medical Center Washington District of Columbia USA

**Keywords:** echocardiography, electrocardiography, hypertension, hypertrophy, left ventricular, prediction

## Abstract

**Background:**

Electrocardiography is the first‐choice technique for detecting left ventricular hypertrophy in patients with arterial hypertension. It is necessary to know the probable outcome for every patient during the treatment, with the aim of improving cardiovascular event prevention.

**Hypothesis:**

Certain electrocardiographic criteria for left ventricular hypertrophy may predict outcomes of patients with left ventricular hypertrophy during a 15‐year follow‐up.

**Methods:**

Fifteen‐year prospective study of 83 consecutive patients (53 male and 30 female; mean age 55.3 ± 8.1) with echocardiographic left ventricular hypertrophy (left ventricular mass index 170.3 ± 31.6 g/m^2^). Electrocardiographic left ventricular hypertrophy was determined by means of Gubner‐Ungerleider voltage, Lewis voltage, voltage of R wave in aVL lead, Lyon‐Sokolow voltage, Cornell voltage and Cornell product, voltage RV_6_ and RV_5_ ratio, Romhilt‐Estes score, Framingham criterion and Perugia criterion.

**Results:**

One or more composite events were registered in 32 (38.5%) patients during 15‐year follow‐up. Positive Lyon‐Sokolow score (17.6% vs. 47.3%; *P* < 0.05), Lewis voltage (9.8% vs. 21.9%; *P* < 0.05), Cornell voltage (15.7% vs. 37.5%; *P* < 0.05), and Cornell product (9.8% vs. 34.4%; *P* < 0.01) were more frequent in a group of patients with composite events. Odd ratio for Cornell product was 4.819 (95% CI 1.486‐15.627).

**Conclusion:**

Patients with echocardiographic left ventricular hypertrophy who had positive Lewis voltage, Lyon‐Sokolow voltage, Cornell voltage, and Cornell product showed worse 15‐year outcome. The strongest predictor of cardiovascular events was positive result of Cornell product.

## 1 INTRODUCTION

Left ventricular mass is measured by accurate techniques, such as echocardiography, computerized tomography, magnetic resonance, and three‐dimensional echocardiography. Electrocardiography (ECG) is the first‐choice technique for detecting left ventricular hypertrophy (LVH) in patients with hypertension due to its wide‐scale availability, low cost, repeatability, and established value prognostic.^1^, ^2^, ^3^ Current guidelines for diagnosis and treatment of hypertension strongly recommend ECG as the only examination to be performed in all hypertensive subjects for detection of LVH.^4^ However, it is well known that majority of LVH are left undetected. The presence of electrocardiographic LVH predicts a several‐fold increase cardiovascular morbidity and mortality in patients with essential hypertension.^5^, ^6^, ^7^ The most effective strategy for cardiovascular event prevention implies extensive knowledge of the probable outcome for each patient during the treatment.

The aim of the paper is to investigate clinical and prognostic significance of electrocardiographic criteria of hypertensive left ventricular hypertrophy during 15‐year follow‐up period.

## 2 METHODS

### 2.1 Population study

This study represented an extension of our previously published study which had analyzed the above stated criteria during 5‐year follow‐up period.^8^ The study was conducted at the Institute for Treatment and Rehabilitation “Niska Banja.” The current study is part of the project “Prognostic significance of non‐invasive parameters at patients with hypertension and left ventricular hypertrophy.” The continuation of a 5‐year research was approved on the session of Ethical Committee of Institute “Niska Banja” held on January 26, 2010. (Resolution No. 3‐676/2). The primary criterion for including patients in the study was the presence of echocardiographic LVH without cardiovascular events. The patients were recruited in the period June 1998 to June 2001. Eighty‐three patients met the criterion for being included in the study (average age 55.3 ± 8.1; 53 male and 30 female). The patients were subjected to 15‐year treatment and monitoring. Composite cardiovascular events occurred in 32 (38.5%) patients during 15‐year follow‐up. The total of 6 (7.2%) patients had myocardial infarction (death occurred in 3 patients—3.6%), 14 (16.9%) patients had cerebrovascular insult (6 patients died—7.2%), 6 (7.2%) patients had positive exercise testing, one patient did not survive coronary revascularization (1.2%), 3 (3.6%) patients died from sudden cardiac death, while 2 (2.4%) patients developed heart failure.

The diagnosis of hypertension, target values of systolic and diastolic blood pressure, pharmacological therapy, exclusion criteria, and verification of adverse events were the same as in our previously published study.^5^

### 2.2 Electrocardiogram and criteria for left ventricular hypertrophy

LVH was determined by means of Gubner‐Ungerleider voltage, Levis voltage, voltage of R wave in aVL lead (positive >1.1 mV), Lyon‐Sokolow voltage, Cornell voltage and Cornell product, voltage RV_6_ and RV_5_ ratio (positive >1), Romhilt‐Estes score, Framingham criterion, and Perugia criterion (electrocardiograph EKG‐300, EI Nis). Additionally, Gubner‐Ungerleider voltage as RD_1_ + SD_3_ ≥ 2.5 mV, and Lewis voltage as (RD_1_ + SD_3_) — (SD_1_+ RD_3_) ≥ 1.7 mV were used for confirming LVH. Positive Lyon‐Sokolow voltage was defined as SV_1_ + RV_5_ or V_6_ ≥ 3.5 mV, or in accordance with European Society of Cardiology Guidelines Committee as ≥3.8 mV.^9^ In line with Cornell voltage criteria, the presence of LVH was defined as SV_3_ + RaVL > 2.0 for women and >2.8 for men. Positive Cornell product was defined as SV_3_ + RaVL × QRS duration ≥244 mV × ms. Left ventricular strain was defined as ST‐segment depression ≥0.1 mV plus T‐wave asymmetric inversion in V_2_ to V_6_ and in peripheral leads (lateral or inferior). Framingham criterion was positive if left ventricular (LV) strain plus ≥1 voltage criterion (RaVL > 1.1 mV, RD_1_ + SD_3_ ≥ 2.5 mV, SV_1_ or V_2_ + RV_5_ or V_6_ ≥ 3.5 mV, SV_1_ or V_2_ ≥ 2.5 mV, RV_5_ or V_6_ ≥ 2.5 mV). Perugia criterion was positive if SV_3_ + RaVL >2.4 mV (men), >2.0 mV (women), and/or LV strain, and/or Romhilt‐Estes score ≥5. Positive Romhilt‐Estes score was defined as ≥5 points and calculated from six ECG features with a specific value of points for each feature: R or S wave in any limb lead ≥2.0 mV or S wave in V_1_ or V_2_ ≥ 3.0 mV or R wave in V_5_ or V_6_ ≥ 3.0 mV (three points); P‐terminal force defined as terminal negativity of P wave in V_1_ ≥ 0.10 mV in depth and ≥0.04 ms in duration (three points); LV strain defined as ST segment and T wave in opposite direction to QRS in V_5_ or V_6_, without digitalis (three points); left axis deviation defined as QRS axis less than or equal to −30° (two points); QRS duration ≥0.09 ms (one point); and intrinsicoid deflection in V_5_ or V_6_ ≥ 0.05 ms (one point).^10^

### 2.3 Detection of left ventricular hypertrophy

Detection of echocardiographic left ventricular hypertrophy was done by means Acuson Sequoia C250 with 3.5 MHz, using M‐mode technique.^8^ Measurements were carried out in accordance with the rules of Penn convention, after which left ventricular mass was calculated.^11^, ^12^ Left ventricular mass was indexed by body surface area, while cut off values for left ventricular mass index were defined as ≥110 g/m^2^ for women and ≥134 g/m^2^ for men.^11^ All details of echocardiographic examination were explained in our previously published study.^8^

### 2.4 Coronary artery disease detection

The first test for detecting coronary artery disease implied treadmill Bruce protocol. Ergometric testing was performed every second year or at more frequent intervals, if necessary (clinically suspected coronary artery disease). If the test data were not sufficient, stress echocardiography was carried out. Patients with positive exercise test (ST depression of ≥1 mm) were subjected to coronary angiography. More details about exercise testing protocol could be seen in our previously published study.^8^

### 2.5 Blood pressure measurement

In addition to continuous blood pressure monitoring in medical office, each patient was subjected to 24‐hour ambulatory blood pressure monitoring (Del Mar Avionics, Irvine, CA equipment, model P‐VA and P6).^5^ Extreme values of blood pressure recorded during 24‐hour measurement were not taken for statistical processing (more details in Djordjevic et al.^8^).

### 2.6 Statistical analysis

Statistical analysis was performed by means of SPSS software (SPSS Inc., version 17.0). Average values and SDs were calculated. Percentages were used for descriptive variables. Differences between groups were tested by ANOVA and *χ* ^2^ tests. Multiple regression analysis and the odds ratio were calculated for all electrocardiographic criteria of left ventricular hypertrophy in terms of presence of cardiovascular events. Receiver operating characteristic curve analysis was performed.

## 3 RESULTS

Statistically, both groups, that is, group with adverse events and group without adverse events, had the same basic characteristics and received the same therapy at the end of the study (Table 1).

**TABLE 1:** Baseline characteristic of examined population and therapy at the end of the study

Characteristics	All (N = 83)	Without AE (N = 51)	With AE (N = 32)
Gender (male/female)	53/30	32/19	21/11
Age (y)	53.3 ± 8.1	55.2 ± 8.1	55.4 ± 8.2
Body mass index (kg/m^2^)	29.1 ± 3.7	29.1 ± 4.0	29.2 ± 3.4
Duration of hypertension (y)	12.1 ± 7.7	12.5 ± 8.5	11.5 ± 6.7
Smoking (number/%)	31/37.3	18/35.3	13/40.6
Cholesterol >5 mmol/L (number/%)	64/77.1	38/74.5	26/81.2
Diabetes mellitus (number/%)	11/13.2	6/11.8	5/ 15.6
Therapy at the end of the study			
Beta‐blockers (number / %)	59/71.1	37/72.6	22/68.7
ACE inhibitors/ARB (number / %)	68/81.9	43/84.3	25/78.1
Calcium channel blockers (number / %)	43/51.8	26/51.0	17/53.1
Diuretics (number / %)	52/62.6	31/60.8	21/67.7

*Note*: Data are mean ± SD.

Abbreviation: AE, adverse events; ACE, angiotensin converting enzyme; ARB, angiotensin receptors blockers.

Parameters obtained by 24‐hour ambulatory blood pressure monitoring and the ones obtained by echocardiography were shown in Table 2. The presented tables showed that there were no statistically significant differences between groups in terms of blood pressure, as well as in terms of echocardiographic parameters.

**TABLE 2:** Parameters of 24‐hour ambulatory blood pressure monitoring and echocardiographic parameters in patients with and without adverse cardiovascular events

Parameters	All (N = 83)	Without AE (N = 51)	With AE (N = 32)
Average 24‐h SBP (mm Hg)	139.0 ± 16.6	141.6 ± 15.7	134.9 ± 17.3
Average 24‐h DBP (mm Hg)	85.8 ± 10.2	87.3 ± 9.3	83.4 ± 11.2
SD SBPD (mm Hg)	15.4 ± 3.9	15.8 ± 3.8	14.7 ± 3.9
SD SBPN (mm Hg)	11.7 ± 4.8	11.2 ± 4.8	12.4 ± 4.8
SD DBPD (mm Hg)	11.8 ± 3.0	12.3 ± 3.2	11.2 ± 2.4
SD DBPN (mm Hg)	10.3 ± 3.3	10.3 ± 3.4	10.2 ± 3.3
PFSBP (%)	7.4 ± 9.6	7.2 ± 9.6	7.8 ± 9.7
PFDBP (%)	9.6 ± 10.8	9.1 ± 11.2	10.5 ± 10.4
LVID (mm)	53.0 ± 4.7	52.9 ± 4.9	53.1 ± 4.5
SWT (mm)	13.6 ± 2.5	14.0 ± 2.3	13.1 ± 2.6
PWT (mm)	11.7 ± 1.2	11.9 ± 1.1	11.5 ± 1.3
LVM (g)	336.2 ± 75.1	345.0 ± 69.7	322.2 ± 82.2
LVMI (g/m^2^)	170.3 ± 31.6	170.7 ± 28.0	169.8 ± 37.1
RWT	0.45 ± 006	0.45 ± 0.06	0.44 ± 0.07
EF (%)	66.0 ± 5.9	65.6 ± 5.3	66.6 ± 6.8
LA (mm)	40.0 ± 5.2	30.7 ± 5.5	39.0 ± 4.6
E/A	1.00 ± 0.29	0.97 ± 0.26	1.06 ± 0.32

*Note*: Data are mean ± SD.

Abbreviations: AE, adverse events; DBP, diastolic blood pressure; E/A, early transmitral velocity/late transmitral velocity; EF, ejection fraction; LA, left atrium; LVID, left ventricular internal dimension; LVM, left ventricular mass; LVMI, left ventricular mass index; PFDBP, percent of fall diastolic blood pressure; PFSBP, percent of fall systolic blood pressure; PWT, posterior wall thickness; RWT, relative wall thickness; SBP, systolic blood pressure; SD DBPD, SD of diastolic blood pressure during the day; SD DBPN, SD of diastolic blood pressure during the night; SD SBPD, SD of systolic blood pressure during the day; SD SBPN, SD of systolic blood pressure during the night; SWT, septal wall thickness.

Both groups had similar functional capacity during exercise testing measured by METs ‐ metabolic equivalents (without adverse events 6.9 ± 2.5 METs vs. with adverse events 6.3 ± 2.2 METs) and by double product (155.1 ± 51.5 vs. 145.7 ± 63.2). Moreover, there was no difference in terms of other parameters obtained by cardiac stress test. The total of 32 (38.5%) patients had eccentric LVH, 31 (37.4%) patients had concentric LVH, and 20 (24.1%) patients had disproportionate septal LVH.

Positive Lyon‐Sokolow score, Lewis voltage and Cornell voltage (Table 3) were more frequent in group with new cardiovascular events as compared to group without cardiovascular events (*P* < 0.05). Positive Cornell product for LVH was statistically significant for patients with cardiovascular events (*P* < 0.01). Multiple regression stepwise analyses highlighted Cornell product (standardized coefficient beta 0.303; *P* < 0.001) as compared to other criteria of hypertrophy, after adjustments in terms of gender, age, body mass index (BMI), and LVMI (model: R 0.303, R^2^ 0.092, adjusted R^2^ 0.081, standardized error of estimate 0.46951).

**TABLE 3:** Odds ratio and distribution of positive electrocariographic parameters in examined groups

Parameters	Odds ratio (95% CI)	Without AE (N = 51)	With AE (N = 32)
		n (%)	n (%)
Gubner‐Ungerleider voltage ≥2.5 mV	1.143 (1.003‐1.304)	0 (0.0%)	4 (12.5%)
Lewis voltage ≥1.7 mV	2.576 (0.740‐8.961)	5 (9.8%)	7 (21.9.0%)*
R aVL > 1.1 mV	1.143 (1.003‐1.303)	0 (0.0%)	4 (12.5%)
R V_5_ or V_6_ ≥ 2.5 mV	3.290 (0.878‐12.326)	4 (7.8%)	7 (21.9%)
S V_1_ or V_2_ ≥ 2.5 mV	7.143 (0.761‐67.069)	1 (2.0%)	4 (12.5%)
Lyon‐Sokolow voltage ≥3.5 mV	3.630 (1.331‐9.896)	9 (17.6%)	14 (43.7%)*
Lyon‐Sokolow voltage ≥3.8 mV	2.500 (0.777‐8.044)	6 (11.8%)	8 (25.0%)
Cornell voltage >2.8 mV (men); >2.0 mV (women)	3.225 (1.140‐9.125)	8 (15.7%)	12 (37.5%)*
Cornell product ≥244 mV × ms	4.819 (1.486‐15.627)	5 (9.8%)	11 (34.4%)**
RV_6_:RV_5_ ratio	0.740 (0.262‐2.093)	14 (27.4%)	7 (21.9%)
Romhilt‐Estes score ≥ 5	2.286 (1.076‐10.963)	3 (5.9%)	4 (12.5%)
Framingham criterion	1.216 (0.254‐5.824)	1 (2.0%)	1 (3.1%)
Perugia criterion	2.488 (0.942‐6.570)	11 (2.0%)	13 (3.1%)

*Note*: Data are mean ± SD.

Abbreviation: AE, adverse events.

* *P* < 0.05.

** *P* < 0.01.

## 4 DISCUSSION

The prevalence of arterial hypertension in Serbia is 42.7%.^13^ Left ventricular hypertrophy has been a well‐known complication of arterial hypertension. It is actually a sign of primary hypertension organ damage and indicates late‐stage of the disease, which is a marker of bad prognosis for the occurrence of cardiovascular and cerebrovascular events. It can be diagnosed by radiography, ECG, echocardiography and, recently, by nuclear magnetic resonance.

The incidence of left ventricular hypertrophy may vary and mostly depends on diagnostic method. The standard ECG is reliable for diagnosing LV hypertrophy, regardless of the presence of atrial fibrillation or sinus rhythm at the time of ECG recording.^14^ The newly proposed ECG criteria (Peguero) are calculated by adding the amplitude of the deepest S wave of D lead in any single lead to the S‐wave amplitude of lead V_4_. The criteria provide an improved sensitivity for the ECG diagnosis of LVH compared to existing criteria.^15^ Peguero ECG LVH is predictive of increased risk of death similar to the traditional ECG‐LVH criteria.^16^

Additionally, prognostic significance of LVH may vary. Leigh et al.^17^ monitored patients aged ≥65 during 10.6 years of follow‐up and found the frequency of ECG‐LHV at 3.5% pts and echocardiography hypertrophy at 11% pts. They concluded that the association of ECG‐LVH with CVD events did not depend on echocardiographic LVH. This finding supported the perception that electrocardiographic LVH was an electrophysiological marker with predictive properties independent of LV anatomy. Similarly, the conclusion of Bacharova et al.^18^ research was that discrepancy in LVH detection by ECG and magnetic resonance imaging could be improved by taking participants' characteristics into consideration. Discrepancy in diagnostic performance and agreement on predictive ability suggested that LVH by ECG and LVH by imaging were likely to be distinct, but related phenotypes. Nuclear magnetic resonance was the most precise method of identifying LVH, but since it was not always available, ECG represented a significant and most available method in daily clinical practice. ECG had low sensitivity and high specificity.^19^

Nowadays, numerous experts attempt to redefine the existing LVH criteria by using magnetic resonance as gold standard. Rodrigues et al.^19^ noticed that obese individuals had less frequent LHV positive results in terms of voltage criterion and suggested their correction. The prognostic significance of this should be tested on large number of patients. Each positive step in terms of increasing sensitivity and preserving specificity had a significant clinical importance for this inexpensive and widely available diagnostic method.^20^ Leese et al.^21^ pointed out that combination of ECG and selective use of echocardiography could reduce the costs in the United States, which was supported by cost‐effectiveness analysis. On the other hand, European guidelines recommended that each patient should be subjected to echocardiography and electrocardiographic diagnosis of left ventricular hypertrophy by means of corrected Lyon‐Sokolow criteria and Cornell product.^9^ This study tested prognostic significance of large number of electrocardiographic criteria during a 15‐year patient treatment and follow‐up. The patients were divided into two groups based on cardiovascular and cerebrovascular events which occurred during the study. All patients were treated in accordance with valid recommendations for treating hypertension and thus there were no statistically significant differences in terms of applied therapy. This was very important, since the evidence showed that the regression of LVH after hypertensive treatment was accompanied by improvement in prognosis.^22^ This study showed no statistically significant differences between groups in terms of risk factors, BMI and blood pressure parameters, despite the fact that these factors might be responsible for the development of left ventricular hypertrophy. Recently published study indicated that visit‐to‐visit blood pressure variability was a predictor of cardiovascular risk category in general population.^23^ Hansen et al.^24^ showed that correlation between 24‐hour ambulatory blood pressure monitoring and LVH was better than the correlation between office‐measured blood pressure and LVH. The values of blood pressure were not good predictors of morbidity and mortality in patients with LVH, as it was shown in our 5‐year study.^4^, ^25^

After completing ergometric test, coronary heart disease was excluded from this study. Patients included in the study showed no difference in stress level or double product. The authors of numerous studies showed that low achieved heart rate, exercise capacity and Duke Treadmill score were dominant predictors of worst prognosis.^26^, ^27^

Even though there were no differences between the tested groups in terms of basic features, medical treatment, blood pressure level, echocardiography, and ergometric parameters, the study showed difference in distribution of electrocardiographic markers of left ventricular hypertrophy. Having in mind the sensitivity of specific criteria and small number of cardiovascular events, which was a limitation of this study, not all known criteria of electrocardiographic left ventricular hypertrophy could undergo a valid statistical analysis. The study of Estes et al.^10^ showed that complicated Romhilt‐Estes criterion for left ventricular hypertrophy was a good prognostic marker during 21.7‐year follow‐up of general population. The score of ≥5 was registered in only 1.4% of patients. Even though the patients included in this study had electrocardiographic left ventricular hypertrophy, it was present with very low percentage in both groups, that is, group without adverse events and group with adverse events (5.9% vs. 12.5%). Complexity and low presence in the first hypertrophy made it unfit for routine application. By using nuclear magnetic resonance as gold standard, Courand et al.^28^ concluded that the voltage of R wave in aVL lead between 0.5 and 1 mV was a strong indicator of left ventricular hypertrophy. Similarly, Rodrigues et al.^29^ recognized that the voltage of R wave in aVL lead ≥0.55 mV had good sensitivity (87%) for detecting LVH with preserved specificity (75%). This study showed no positive results of LVH based on R wave voltage (> 1.1 mV) in aVL lead at patients without adverse events, while it was present in 12.5% patients with composite events. However, the study highlighted four electrocardiographic criteria (Lewis voltage, Lyon‐Sokolow voltage, Cornell voltage, and Cornell product) which were present in a group of patients with new composite events and thus were statistically significant. In the study of Levy et al.,^30^ Lyon‐Sokolow score and Cornell voltage QRS duration product were independent predictors of adverse cardiovascular events. The study of Tan et al.^31^ concluded that the Cornell product was easily applicable ECG marker of heart failure with preserved ejection fraction and predicted poor outcome by emphasizing the severity of diastolic dysfunction and LV hypertrophy. In the study of O'Neal et al.,^32^ the authors concluded that electrocardiographic and echocardiographic LVH could be used interchangeably in stroke risk scores (Harrell's concordance index). The study of Japanese Trial to Assess Optimal Systolic Blood Pressure in Elderly Hypertensive Patients (JATOS) found that ECG LVH was strongly related to cardiovascular events in elderly hypertensive patients.^33^ The study showed that hazard ratio for cardiovascular events was 2.17 (95% CI: 1.54‐3.05, *P* < 0.0001) and 2.83 (95% CI: 1.91‐4.19, *P* < 0.0001) when SV_1_ + RV_5_ was classified into two groups at threshold values of 35 and 40 mm, respectively. In terms of analyzing Lyon‐Sokolow voltage, odds ratio in this study was 3.630 (1.331‐9.896; *P* < 0.05) for composite cardiovascular events. Losartan Intervention for Endpoint Reduction in Hypertension (LIFE) Study proved that the reduction of electrocardiogram hypertension resulted in the reduction of cardiovascular risk, regardless of the changes in blood pressure.^34^

## 5 CONCLUSION

Positive Lewis voltage, Lyon‐Sokolow voltage, Cornell voltage and Cornell product showed worse 15‐year outcome in patients with hypertensive heart. The strongest predictor of cardiovascular events was positive result of Cornell product.

## CONFLICT OF INTEREST

The authors certify that they have no affiliations with or involvement in any organization or entity with any financial interest, or non‐financial interest, such as personal or professional relationships, affiliations, knowledge or beliefs in the subject matter or materials discussed in this manuscript.
